# Triple-negative breast cancer is associated with EGFR, CK5/6 and c-KIT expression in Malaysian women

**DOI:** 10.1186/1472-6890-12-18

**Published:** 2012-09-26

**Authors:** Shant Kishen Kanapathy Pillai, Annie Tay, Suseela Nair, Chee-Onn Leong

**Affiliations:** 1 School of Medicine, International Medical University, Bukit Jalil, Kuala Lumpur, 57000, Malaysia; 2 Gleneagles Hospital Kuala Lumpur, 282 & 286 Jalan Ampang, Kuala Lumpur, 50450, Malaysia; 3 School of Pharmacy and Health Sciences, International Medical University, Bukit Jalil, Kuala Lumpur, 57000, Malaysia; 4 International Medical University, 126 Jalan 19/155B, Bukit Jalil, Kuala Lumpur, 57000, Malaysia

**Keywords:** Triple-negative breast cancers, Basal-like, EGFR, CK5/6, c-Kit

## Abstract

**Background:**

Triple-negative breast cancer (TNBC) is a heterogeneous subgroup of breast cancer characterized by the lack of estrogen receptor (ER), progesterone receptor (PR) and the human epidermal growth factor receptor 2 (HER2) expressions. This subgroup of refractory disease tends to have aggressive clinical behavior, high frequency of metastasis and lack of response to current hormonal or targeted therapies. Despite numerous studies reporting the clinicopathological features of TNBC and its association with the basal-like phenotype in the Western population, only limited data are available in the Asian population. Therefore, the aim of this study was to investigate the clinicopathological characteristics of TNBC and its association with epidermal growth factor receptor (EGFR), cytokeratin 5/6 (CK5/6) and mast/stem cell growth factor receptor (c-KIT or CD117) expression in Malaysian women.

**Methods:**

A total of 340 patients diagnosed with primary breast cancer between 2002 and 2006 in Malaysia were reviewed and analyzed.

**Results:**

The incidence of TNBC was 12.4% (42/340). Bivariate analysis revealed that TNBC was strongly associated with a younger age, higher grade tumor and p53 expression. Further immunohistochemical analysis suggested that TNBC in Malaysian women was strongly associated with EGFR, CK5/6 and c-KIT expression with high a Ki-67 proliferation index.

**Conclusion:**

In conclusion, our study confirms the association of TNBC with basal-like marker expression (EGFR, CK5/6 and c-KIT) in Malaysian women, consistent with studies in other populations.

## Background

The recent advances of DNA microarray technology has enabled the classification of breast cancer into subgroups based on the gene expression profile [1]. Based on the study of these profiles, breast cancer can be divided into five subtypes: luminal A, luminal B, basal-like, normal-like and human epidermal growth factor receptor 2 (HER2)-overexpressing subtype [1-3]. Of particular importance is the basal-like subtype, which accounts for 15 to 20% of all breast cancers and confers a markedly poor prognosis compared to other subtypes [1,4,5].

The majority of basal-like breast cancers exhibit a “triple-negative” phenotype, characterized by the lack of expression of the estrogen receptor (ER) or the progesterone receptor (PR) and a lack of HER2 amplification. They also often have a high frequency of p53 mutations [6,7].

In most cases, basal-like breast cancer is conveniently defined based on the “triple-negative” phenotype. However, there is evidence that triple-negative breast cancers (TNBC) and basal-like breast cancers (BLBC) might be of two different biological entities [8-10]. Indeed, it has been reported that only 80% of the tumor that express basal-like markers (EGFR, CK5/6 and/or c-KIT) are triple-negative in a Western population [1,4,8,11-13].

Although the clinicopathologic characteristics of the basal-like carcinomas, compared with other subtypes, have been reported in the Korean and Singaporean populations recently [14,15], the true relationship between triple-negative breast cancer and those showing basal-like expression markers has not been enunciated. Thus, our study aimed to investigate the pathology of TNBC in Malaysian women and comprehend the relationship between TNBC and BLBC in our population.

## Methods

### Tissue and patient data

Patients diagnosed with primary breast cancer at the Gleneagles Intan Medical Centre (GIMC), Malaysia, between 2002 and 2006 were included in the study. Clinicopathological parameters including age, tumor size, histological grade, histological subtype, associated ductal carcinoma in situ (DCIS), lymphovascular invasion and nodal status were evaluated. ER and PR statuses were determined using a standard immunohistochemistry (IHC) staining protocol on initial diagnostic material using proteinase K antigen retrieval method followed by mouse anti-human ERα monoclonal antibody (clone 1D5; DAKO, Denmark) and mouse anti-human PR monoclonal antibody (clone PgR 636; DAKO, Denmark). ER or PR positivity was defined as the presence of 1% or more positively-stained tumor cells as described previously [16,17]. HER2 expression was determined using the DAKO Herceptest® Kit (Dako, Carpinteria, CA, USA) and scored according to international guidelines [18]. HER2 scores of 0 and 1+ were considered negative. HER2 scores of 2+ and 3+ were considered as HER2 overexpression [16]. All results were available from the original pathology reports except for HER2 amplification which was not determined at the time of diagnosis. Triple-negative breast cancers (TNBC) were defined as tumors that were ER, PR and HER2 negative. Non triple-negative (non-TNBC) cases were defined as tumors that express ER, PR or HER2. This study was approved by the Institutional Review Board of the International Medical University, Malaysia (IRB protocol number IMU-BMS I02/2009-2). Written informed consent for use of all human specimens in this study was obtained at the time of enrollment.

### Immunohistochemistry (IHC)

Immunohistochemistry (IHC) analysis of human epidermal growth factor receptor (EGFR), cytokeratin 5/6 (CK5/6), mast/stem cell growth factor receptor (c-KIT or CD117) and Ki-67 was performed on formalin-fixed, paraffin-embedded breast cancer tissue. Tissue blocks were sectioned at 4-μm thickness and deparaffinized in xylene and rehydrated with graded ethanol. Heat-induced epitope retrieval in Tris/EDTA pH 9.0 buffer was used for CK5/6, c-KIT and Ki-67 staining, while proteinase K enzymatic retrieval method was used for EGFR staining. All primary antibodies were supplied by Dako Corporation (Carpinteria, CA, USA). The dilution factors were as follows: EGFR (clone E30), 1:50; CK5/6 (clone D5/16 B4), 1:50; cKIT polyclonal, 1:400; and Ki-67 (clone MIB-1), 1:100. EGFR, CK5/6 or c-KIT positivity was defined as the presence of 1% or more positively-stained tumor cells as described previously [8,19].

### Determination of proliferation indices

To estimate the growth rate of tumors, the percentage of tumor cells expressing the proliferation marker Ki-67 was measured. A proliferation index was calculated for each tumor lesion by counting the total number of tumor cell nuclear profiles and the number of Ki-67-positive nuclear profiles in randomly and systematically selected fields as described previously [20-22]. On average, 500 nuclear profiles were counted per tumor lesion.

### Statistical analysis

The Fisher’s exact test was used to analyze the correlation between the triple-negative phenotype and EGFR, CK5/6 or c-KIT expression. The Student’s t-test and Mann–Whitney test was used to compare the Ki-67 proliferaton index of TNBC and non-TNBC. All statistical analyses were performed using SPSS for Windows (Version 11). A *P* value of less than 0.05 was considered statistically significant.

## Results

### Triple-negative breast cancer is associated with a younger age and high tumor grade in Malaysian women

A total of 340 breast cancer patient records obtained from the Gleneagles Hospital, Malaysia, from 2002 to 2006 were reviewed and analyzed. The majority of patients were middle aged between 41 and 65 years old, and had a mean age of 49.4 ± 10.4 years. The median age was 48 years. Most of the cases were invasive ductal carcinoma (IDC), accounting for 78.5% of all cases. The majority of these patients also presented with a grade 2 or 3 tumor. The tumor size had a mean of 2.6 ± 1.4 cm and a median of 2.2 cm. Lymph node infiltration and p53 expression were not common in this cohort of patients. The clinical pathological status of the investigated cohort is summarized in Table 1.

**Table 1:** Summary of the clinical pathological status of triple-negative cases (TNBC) and non triple-negative cases (non-TNBC)

**Characteristic**		**No. of patients (%)**	
	**TNBC (N = 42)**	**non-TNBC (N =298)**	**TOTAL**
**AGE AT DIAGNOSIS (y)**			
≤ 40	15 (35.7%)	49 (16.4%)	64 (18.8%)
41 ≤ y ≤ 49	13 (31.0%)	113 (37.9%)	126 (37.1%)
50 ≤ y ≤ 65	13 (31.0%)	107 (35.9%)	120 (35.3%)
> 65	1 (2.4%)	29 (9.7%)	30 (8.8%)
**HISTOLOGY**			
DCIS	3 (7.1%)	35 (11.7%)	38 (11.2%)
IDC	35 (83.3%)	232 (77.9%)	267 (78.5%)
Others	4 (9.5%)	31 (10.4%)	35 (10.3%)
**GRADE**			
1 (well differentiated)	0 (0.0%)	7 (2.3%)	7 (2.1%)
2 (moderately differentiated)	6 (14.3%)	110 (36.9%)	116 (34.1%)
3 (poorly differentiated)	32 (76.2%)	151 (50.7%)	183 (53.8%)
Not determined	4 (9.5%)	30 (10.1%)	34 (10.0%)
**TUMOR SIZE (cm)**			
≤ 2	12 (28.6%)	109 (36.6%)	121 (35.6%)
2 < cm ≤ 5	15 (35.7%)	99 (33.2%)	114 (33.5%)
> 5	3 (7.1%)	15 (5.0%)	18 (5.3%)
Not determined	12 (28.6%)	75 (25.2%)	87 (25.6%)
**LYMPH NODE INFILTRATION**			
Positive	17 (40.5%)	91 (30.5%)	108 (31.8%)
Negative	25 (59.5%)	194 (65.1%)	219 (64.4%)
Not determined	0 (0.0%)	13 (4.4%)	13 (3.8%)
**p53 STATUS**			
Positive	15 (35.7%)	45 (15.1%)	60 (17.6%)
Negative	27 (64.3%)	251 (84.2%)	278 (81.8%)
Not determined	0 (0.0%)	2 (0.7%)	2 (0.6%)

Note: Percentage (%) indicates the percentage within the subgroup. *DCIS* ductal carcinoma in-situ, *IDC* invasive ductal carcinoma.

All cases were further stratified based on ER, PR and HER2 statuses. A total of 42 cases (12.4%) were identified to be TNBC and the remaining 298 cases (87.6%) expressed at least one of the markers and were classified as non-TNBC cases. Among all the non-TNBC cases, a total of 111 (37.2%) cases were ER/PR + and HER2+, 106 (35.6%) cases were ER/PR + and HER2-, and 81 (27.2%) cases were ER/PR- and HER2+ (Table 2). Of note, the majority of patients diagnosed with TNBC were of a younger age (below 40 years) with a mean age of 45.3 ± 10.3 years versus 50.0 ± 10.4 years in the non-TNBC cases (Student’s t-test, *P* = 0.0029). In addition, most of the TNBC cases were high grade tumors with 76.2% of the cases diagnosed as grade 3 versus 50.7% in the non-TNBC group.

**Table 2:** Summary of clinical pathologic status of non triple-negative cases (non-TNBC)

**Characteristic**		**No. of patients (%)**	
	**ER/PR+, HER2+ (N = 111)**	**ER/PR+, HER2- (N =106)**	**ER/PR-, HER2+ (N =81)**
**AGE AT DIAGNOSIS (y)**			
≤ 40	21 (18.9%)	17 (16.0%)	11 (13.6%)
41 ≤ y ≤ 49	42 (37.8%)	45 (42.5%)	26 (32.1%)
50 ≤ y ≤ 65	37 (33.3%)	33 (31.1%)	37 (45.7%)
> 65	11 (9.9%)	11 (10.4%)	7 (8.6%)
**HISTOLOGY**			
DCIS	14 (12.6%)	13 (12.3%)	8 (9.9%)
IDC	89 (80.2%)	74 (69.8%)	69 (85.2%)
Others	8 (7.2%)	19 (17.9%)	4 (4.9%)
**GRADE**			
1 (well differentiated)	2 (1.8%)	4 (3.8%)	1 (1.2%)
2 (moderately differentiated)	50 (45.0%)	48 (45.3%)	12 (14.8%)
3 (poorly differentiated)	52 (46.8%)	35 (33.0%)	64 (79.0%)
Not determined	7 (6.3%)	19 (17.9%)	4 (4.9%)
**TUMOR SIZE (cm)**			
≤ 2	46 (41.4%)	44 (41.5%)	19 (23.5%)
2 < cm ≤ 5	32 (28.8%)	27 (25.5%)	40 (49.4%)
> 5	7 (6.3%)	2 (1.9%)	6 (7.4%)
Not determined	26 (23.4%)	33 (31.1%)	16 (19.8%)
**LYMPH NODE INFILTRATION**			
Positive	34 (30.6%)	24 (22.6%)	33 (40.7%)
Negative	75 (67.6%)	74 (69.8%)	45 (55.6%)
Not determined	2 (1.8%)	8 (7.5%)	3 (3.7%)
**p53 STATUS**			
Positive	10 (9.0%)	7 (6.6%)	28 (34.6%)
Negative	101 (91.0%)	97 (91.5%)	53 (65.4%)
Not determined	0 (0.0%)	2 (1.9%)	0 (0.0%)

Note: Percentage (%) indicates the percentage within the subgroup. *DCIS* ductal carcinoma in-situ, *IDC* invasive ductal carcinoma.

Although the tumor size from the TNBC cases were slightly larger (2.8 ± 1.6 cm) compared to non-TNBC cases (2.5 ± 1.4 cm), the difference, however, was not statistically significant (Student’s t-test, *P* = 0.153). Similarly, no differences in histology (IDC vs DCIS) (Fisher’s exact test, *P* = 0.322) and lymph node infiltration rate (Fisher’s exact test, *P* = 0.177) were observed between TNBC and non-TNBC cases. Thus, the major differences between TNBC and non-TNBC were age and tumor grade, in which TNBC patients were younger and with high grade tumors compared to non-TNBC patients.

### Triple-negative breast cancer is strongly associated with EGFR, CK5/6 and/or c-KIT expression

Based on the available clinical data, tissue samples from a total of 36 patients were reviewed and retrieved for EGFR, CK5/6 and c-KIT staining. Of the 36 samples, 18 were TNBC and 18 were non-TNBC based on the prior ER, PR and HER2 staining. All cases were age and grade matched as closely as possible and the majority was grade 3 tumors. The clinical pathological features of the cases included in the current study are summarized in Table 3.

**Table 3:** Summary of the clinical pathological status of TNBC and non-TNBC used for EGFR, CK5/6 and c-KIT immunohistochemical analysis

**Characteristic**	**No. of patients (%)**	
	**TNBC (N = 18)**	**non-TNBC (N = 18)**
**AGE AT DIAGNOSIS (y)**		
≤ 40	3 (16.6%)	2 (11.1%)
41 ≤ y ≤ 49	10 (55.5%)	7 (38.9%)
50 ≤ y ≤ 65	5 (27.7%)	6 (33.3%)
> 65	0 (0.0%)	3 (16.7%)
**GRADE**		
1 (well differentiated)	0 (0%)	0 (0%)
2 (moderately differentiated)	3 (16.6%)	5 (27.8%)
3 (poorly differentiated)	15 (83.3%)	13 (72.2%)
**TUMOR SIZE (cm)**		
≤ 2	7 (38.9%)	5 (27.8%)
2 < cm ≤ 5	5 (27.8%)	9 (50.0%)
> 5	3 (16.7%)	1 (5.6%)
Unknown	3 (16.7%)	3 (16.7%)
**LYMPH NODE INFILTRATION**		
Positive	4 (21.1%)	8 (44.4%)
Negative	14 (77.8%)	10 (55.6%)

Note: Percentage (%) indicates the percentage within the subgroup.

**Figure 1:**
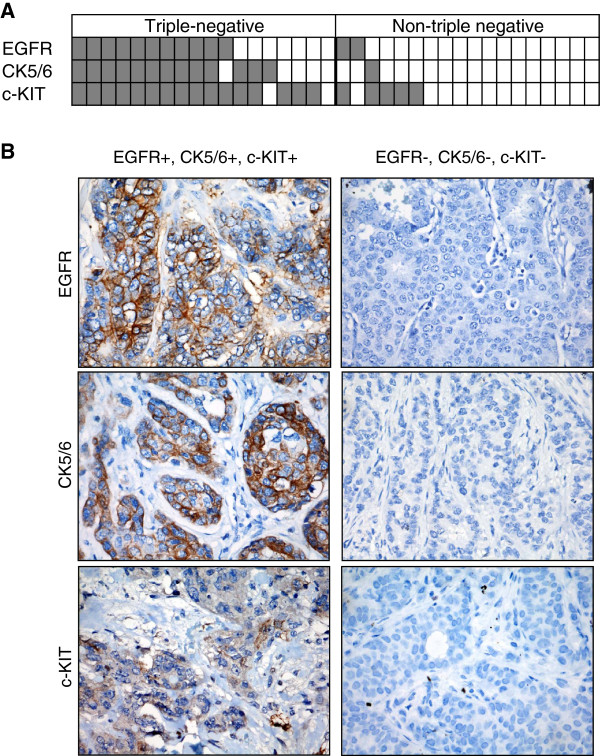
**EGFR, CK5/6 and c-KIT expression is associated with TNBC.** (**A**) A total of 18 TNBC and 18 non-TNBC were stained for EGFR, CK5/6 and c-KIT by IHC. (**B**) Representative immunostaining results for tumors that are EGFR, CK5/6 and c-KIT positive or negative.

Of all the TNBC cases, 61% (11/18) were EGFR+, 72% (13/18) were CK5/6+ and 89% (16/18) were c-KIT+. In stark contrast, only 11% (2/18) were EGFR+, 6% (1/18) were CK5/6+ and 28% (5/18) were c-KIT + in the non-TNBC group (Figure 1 and Table 1). Furthermore, 56% (10/18) of the TNBC cases were both EGFR + and CK5/6+, while none of the non-TNBC cases exhibited co-expression of these markers. Thus, EGFR, CK5/6 or c-KIT expression is strongly associated with TNBC in Malaysian women (Fisher’s exact test, *P* < 0.01) (Table 4).

**Table 4:** Association of TNBC with EGFR, CK5/6 and/or c-KIT expression

**IHC Staining**	**TNBC (%)**	**Non-TNBC (%)**	**Fisher’s exact test (P value)**
**EGFR**			
Positive	11 (61.1%)	2 (11.1%)	4.5 x 10^-5^
Negative	7 (38.9%)	16 (88.9%)	
**CK 5/6**			
Positive	13 (72.2%)	1 (5.6%)	8.3 x10^-3^
Negative	5 (27.8%)	17 (94.4%)	
**c-KIT**			
Positive	16 (88.9%)	5 (27.8%)	4.9 x10^-5^
Negative	2 (11.1%)	13 (72.2%)	
**EGFR and/or CK 5/6**			
Positive	14 (77.8%)	3 (16.7%)	6.1 x10^-4^
Negative	4 (22.2%)	15 (83.3%)	
**EGFR and/or CK 5/6 or c-KIT**			
Positive	17 (94.4%)	6 (33.3%)	3.0 x10^-4^
Negative	1 (5.6%)	12 (66.7%)	

### Triple-negative breast cancers have higher Ki-67 indices

**Figure 2:**
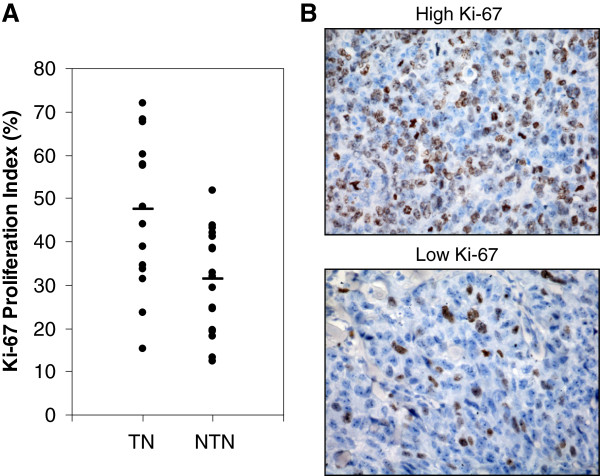
**TNBC has a higher growth rate.** (**A**) Ki-67 proliferation index was used to estimate the growth rate of tumors (16 TNBC and 18 non-TNBC). ·, Ki-67 proliferation index of each tumor; —, the median Ki-67 proliferation index in the TNBC and non-TNBC subgroup. On average, 500 nuclear profiles were counted per tumor lesion. (**B**) Representative immunostaining of tumors that have a high or low Ki-67 proliferation index.

To further characterize the phenotypes of breast cancers in Malaysian women, we also analyzed the Ki-67 proliferation index in TNBC and non-TNBC cases in the current cohort. Thirty six out of 38 specimens (16 TNBC and 18 non-TNBC cases) were stained with a Ki-67-specific antibody (clone MIB-1) and the proliferation index was estimated as the percentage of Ki-67-positive nuclear profiles in randomly and systematically selected fields. As shown in Figure 2, TNBC had a significantly higher Ki-67 index than non-TNBC tumors in Malaysian women (Student’s t-test, *P* = 0.003 and Mann-Whitey test, *P* < 0.004). The mean proliferation index for TNBC and non-TNBC tumors were 47.48 ± 17.55 and 31.43 ± 11.81, respectively. The median proliferation index was 45.98 and 32.39 for TNBC and non-TNBC, respectively. These results suggest that TNBC has a higher proliferation rate than non-TNBC in Malaysian women.

## Discussion

Breast cancer is a heterogeneous group of disease that can be characterized into clinically, morphologically and biologically distinct subgroups [14,23]. By gene expression profiling and IHC markers, breast cancers can be classified into five major subtypes: luminal A (ER + and/or PR+, HER2-), luminal B (ER + and/or PR+, HER2+), HER2-overexpressing (ER-, PR-, HER2+), basal-like (ER-, PR-, HER2-, CK5/6+ and/or EGFR+) and normal breast-like tumors [1,8,12,24,25]. Of particular importance is the basal-like subtype, which accounts for 15 to 20% of all breast cancers and confers a markedly poor prognosis [1,4,5]. Recent studies have shown that basal-like breast cancers are likely to be mitotically active high-grade invasive tumors that are associated with a younger patient age [4,26,27]. A population-based study also identified this subtype to be more prevalent in premenopausal African American women and highly associated with BRCA-1 mutation [4,12,26,27]. Due to their lack of ER, PR and HER2 expression, basal-like breast cancers are also unlikely to respond to anti-estrogen hormonal therapies or trastuzumab [26,28].

To date, the gold standard for identifying basal-like breast cancers is based on gene expression profiling. However, cost and technical issues have rendered gene expression profiling impractical as a routine diagnostic tool in the clinical setting. In the Western population, approximately 70 to 90% of “triple-negative” breast cancers (ER-, PR-, HER2-) express basal markers, resulting in the triple-negative subtype commonly used as a surrogate marker for the basal-like subtype [1,4,8,18,29-37]. Relatively little is known about this disease entity within Asian populations, and in particular Malaysian populations.

Within the small cohort of 340 breast cancer patients described in this study, a total of 42 cases (12.4%) were identified as triple-negative. This proportion was slightly lower than the recent studies in the Malaysian, Japanese, Chinese and Korean populations that estimated the prevalence of TNBC to be around 15 to 20% [23,38-41]. Consistent with earlier studies, our results showed that TNBC in Malaysian women was strongly associated with a younger age and high grade tumors compared to non-TNBC [5,10,14,15,38,42]. However, no significant differences in tumor size, histology (IDC vs DCIS) and lymph node infiltration rates were observed between TNBC and non-TNBC in the current study.

Further analysis was carried out to investigate the expression of EGFR, CK5/6 and c-KIT in TNBC and non-TNBC specimens. Due to the lack of information on HER2 amplification, only tumors with HER2 scores of 0 were included in the TNBC cohort. Our results demonstrated that TNBC in Malaysian women was strongly associated with EGFR, CK5/6 and c-KIT expression. Our results also showed that TNBC had a significantly higher Ki-67 proliferation index than non-TNBC, suggesting that TNBC could be more progressive.

Numerous studies have also shown that basal-like breast cancer can be specifically identified using IHC surrogate panels including ER, PR and HER2 negativity and either EGFR or CK5/6 positivity (ER-, PR-, HER2-, CK5/6+ and/or EGFR+) [8,19,26,43,44]. Using such surrogates, our study showed that 78% (14/18) of TNBC can be categorized as basal-like breast cancers. This proportion is consistent with previous studies that also show that 71.5% of TNBC are basal-like by gene expression profiling [30].

## Conclusions

In conclusion, the incidence of TNBC in this small cohort, predominantly Asian population, is comparable to reported data in other populations. Consistent with other studies, TNBC in Malaysian women is associated with a younger age and higher grade of tumor, as well as p53 expression in bivariate analysis. Our findings also confirm that TNBC in Malaysian women strongly correlates with EGFR, CK5/6 and c-KIT expression, and have a higher proliferation rate.

## Competing interests

The authors declare that they have no competing interests.

## Authors’ contributions

SKKP carried out the IHC staining and data analysis. AT and SN participated in data acquisition and interpretation, and critically revising the manuscript. LCO conceived of the study, and participated in its design and coordination. All authors read and approved the final manuscript.

## Pre-publication history

The pre-publication history for this paper can be accessed here:

http://www.biomedcentral.com/1472-6890/12/18/prepub
